# Supplementary figures and images for: Isoprene-Emitting Tobacco Plants Are Less Affected by Moderate Water Deficit under Future Climate Change Scenario and Show Adjustments of Stress-Related Proteins in Actual Climate
Source: Plants (Basel). 2023 Jan 11;12(2):333. doi: 10.3390/plants12020333 (PMC9862500; doi:10.3390/plants12020333)

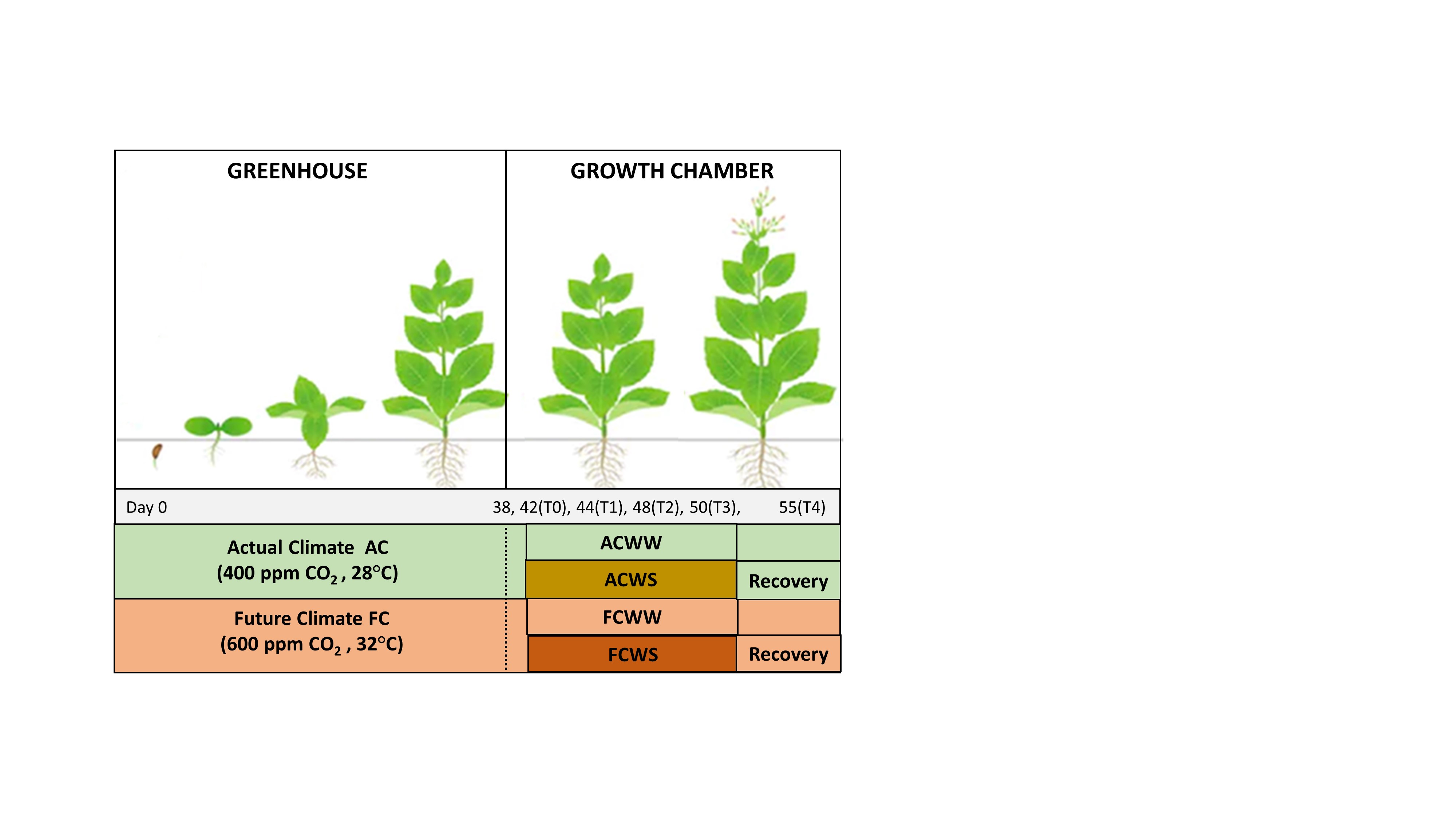

Supplement: Supplementary file 1 [file plants-12-00333-s001.zip › Figure S1.JPG]

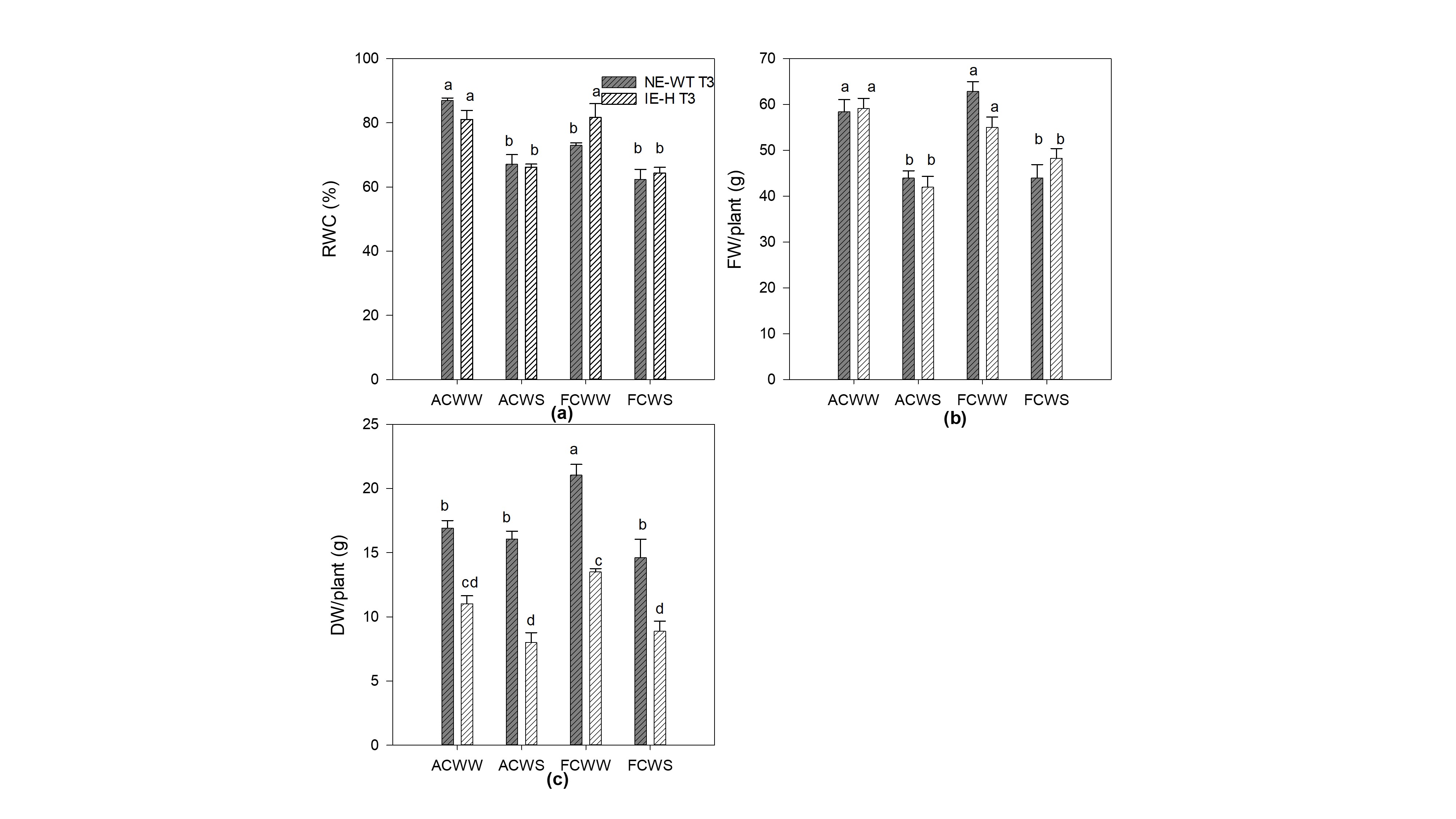

Supplement: Supplementary file 1 [file plants-12-00333-s001.zip › Figure S2.JPG]
